# Endophytic Fungal Diversity in *Carpesium lipskyi* from the Gaoligong Mountains, Yunnan, China

**DOI:** 10.3390/jof11100704

**Published:** 2025-09-28

**Authors:** Hancaiyuan Zheng, Qun Liu, Yining Di, Tao Liu, Yu Su, Yuqin He, Juntong Chen, Jingyi Peng, Shiou Yih Lee, Inh Thkim Hoa, Xianhan Huang, Lufeng Liu

**Affiliations:** 1 College of Resources and Environment, Yunnan Agricultural University, Kunming 650201, China; zhcy2000@163.com (H.Z.);; 2 College of Food, Drug and Health, Yunnan Vocational and Technical College of Agriculture, Kunming 650031, China; 3 State Key Laboratory of Plant Diversity and Specialty Crops, Kunming Institute of Botany, Chinese Academy of Sciences, Kunming 650201, China; 4 College of Agronomy and Biotechnology, Yunnan Agricultural University, Kunming 650201, China; 5 Faculty of Health and Life Sciences, INTI International University, Nilai 71800, Malaysia; 6 Faculty of Health Sciences, Shinawatra University, Samkhok 12160, Thailand; 7 College of Agriculture and Forestry, Thai Nguyen University (TUAF), Conscious Thai Nguyen 250000, Vietnam; 8 State Key Laboratory for Conservation and Utilization of Bio-Resources in Yunnan, Kunming 650201, China

**Keywords:** diversity, genetic resources, high-throughput sequencing, medicinal plants, species richness

## Abstract

Endophytic fungi represent key microbial symbionts that colonize internal plant tissues without causing apparent disease, playing vital roles in host growth, stress resistance, and biosynthesis of bioactive compounds. *Carpesium lipskyi* C. Winkl., a medicinal plant endemic to the Gaoligong Mountains in Yunnan, remains largely unexplored regarding its endophytic fungal composition. In this study, a total of 737 amplicon sequence variants (ASVs) were identified through high-throughput sequencing, spanning 9 phyla, 36 classes, 67 orders, 137 families, 206 genera, and 277 species. The dominant phyla were Ascomycota, Basidiomycota, and Glomeromycota. Alpha diversity in stems and leaves followed a unimodal distribution along the elevational gradient, in contrast to root endophytic communities, which showed no significant correlation with altitude. Peak diversity occurred at 2734 m, indicating a non-linear altitude-diversity relationship. Altitude, along with stable precipitation and temperature (2600–3210 m), significantly influenced fungal diversity. Medicinal fungi such as *Cladosporium sp.*, *Meyerozyma guilliermondii*, *Phialocephala fortinii*, and *Rhodotorula mucilaginosa* were found in either roots or stems. This is the first comprehensive assessment of endophytic fungi in *C. lipskyi* from this region, providing a foundation for future ecological and pharmacological studies.

## 1. Introduction

All plants in natural ecosystems appear to be symbiotic with fungal endophytes. These extremely diverse fungi significantly influence plant communities by enhancing fitness through improved tolerance to abiotic and biotic stresses, increasing biomass, and reducing water use. Alternatively, they may reduce fitness by modifying resource allocation patterns [1]. Among them, endophytic fungi play a vital role in plant growth and development by enhancing water and nutrient uptake, increasing tolerance to environmental stresses, and mediating other synergistic mechanisms [2,3]. In addition, these fungi are also capable of producing a wide range of bioactive compounds with physicochemical properties comparable to those synthesized by plants. This significantly expands the medicinal potential of fungal secondary metabolites and contributes to the chemical and biological diversity of their host plants [4].

As such, research on plant-associated endophytic fungi and their functional capacities has received growing attention. It is estimated that plants can harbor millions of culturable and unculturable endophytic microorganisms, with their diversity and abundance influenced by host species, genotype, and environmental conditions [5,6]. Secondary metabolites produced by specific endophytic fungi have been implicated in enhancing plant resistance to diseases, insects, and abiotic stresses such as drought [7]. Moreover, these fungi often form mutualistic relationships with their hosts, improving the plants’ adaptive capabilities and ecological competitiveness. Through the biosynthesis of phytohormones such as indole-3-acetic acid (IAA) and gibberellins, endophytic fungi can regulate plant physiological processes and support normal growth and metabolism under adverse conditions [8].

Environmental factors are key drivers shaping the community composition and diversity of endophytic fungi [9]. Notably, altitudinal gradients exert a strong influence on the distribution patterns and species richness of these fungi, primarily by inducing changes in temperature, humidity, precipitation, and soil properties. Along these elevation gradients, fungal alpha diversity typically follows a unimodal pattern: peaking at mid-elevations and declining at higher altitudes, where harsher climatic conditions constrain fungal survival and reproduction [10]. Endophytic fungi often display distinct responses across different plant organs, a pattern arising from variations in microenvironments and host functional traits. Moreover, climatic stability, particularly the degree of seasonal variation, governs the adaptation and community assembly of fungal endophytes [11]. Despite extensive research on plant endophytic fungi and their environmental interactions, the genus *Carpesium* L. has received little attention.

*Carpesium* L., a genus in the family Asteraceae (tribe Inuleae, subtribe Inulinae), is primarily distributed in Central Asia. To date, 21 species have been reported worldwide, with 17 species and three varieties recorded in China, especially prevalent in the mountainous regions of southwestern China. *Carpesium lipskyi* C. Winkl. is a Chinese endemic species, found mainly in Gansu, Qinghai, Sichuan, and Yunnan provinces, growing at elevations between 2000 and 3500 m in forest margins and shrublands [12]. The dried whole plant has been traditionally used in Chinese folk medicine to treat ailments such as sore throat, skin ulcers, stomach pain, and snake or insect bites [13]. Given its unique ecological habitat, *C. lipskyi* may harbor a diverse and unique community of endophytic fungi [14].

However, there is limited knowledge regarding the diversity and composition of endophytic fungi associated with *C. lipskyi*, particularly in the Gaoligong Mountains region of Yunnan. Therefore, the present study aims to investigate the endophytic fungal communities of *C. lipskyi* across different elevations in the Gaoligong Mountains using high-throughput sequencing. The results of this study are expected to further facilitate the exploration of endophytic fungal resources in *Carpesium* plants in this region and provide a foundation for subsequent application-oriented research.

## 2. Materials and Methods

### 2.1. Plant Materials and Sampling Site Information

**Figure 1:**
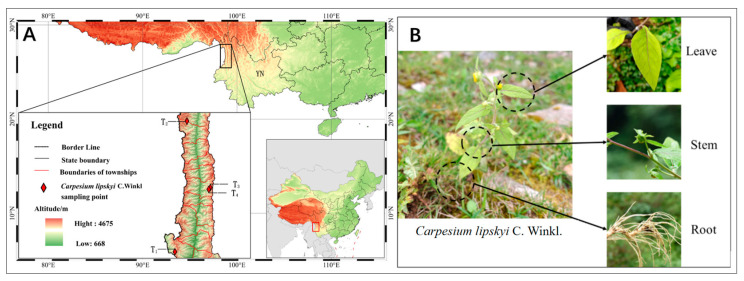
The diagram of the sample collection sites and plant structure: (**A**) schematic diagram of the collection points of *Carpesium lipskyi*, (**B**) schematic diagram of the structure of the *Carpesium lipskyi* model. The map data used in this study was sourced from https://www.geoboundaries.org and https://www.gebco.net (accessed on 16 August 2024).

According to the *Flora of China*, the flowering period of the genus *Carpesium* typically occurs from June to August each year. Considering the unique alpine environment of the Gaoligong Mountains, sample collection was conducted in August 2023 across *Carpesium* populations located at different sites and elevations in the Gaoligong Mountains and Changchong Mountain in Kunming, Yunnan Province (Table 1, Figure 1). For each population, four biological replicates were collected. Each plant sample was divided into three parts—roots, stems, and leaves—which were cut into 3–5 cm segments [15]. In the field, surface sterilization was performed promptly using 75% ethanol for 1 min, followed by 2.5% sodium hypochlorite for 5 min. Samples were then rinsed 2–3 times with sterile water, stored in sterile centrifuge tubes, and preserved in liquid nitrogen. Additionally, one specimen per population was retained and prepared as a voucher specimen to facilitate future taxonomic identification. At the end of the sampling period, all samples were transferred to a −80 °C ultra-low temperature freezer at the Kunming Institute of Botany, Chinese Academy of Sciences.

### 2.2. Genomic DNA Extraction and PCR Amplification

Genomic DNA was extracted from samples using the Omega DNA Kit (M5635-02; Omega Bio-Tek, Norcross, GA, USA), following the manufacturer’s protocol. DNA quality and concentration were assessed using NanoDrop NC2000 (Thermo Fisher Scientific, Waltham, MA, USA) and agarose gel electrophoresis. Extracted DNA was stored at −20 °C until further analysis.

Briefly, 0.8 mL of Buffer SLX Mlus was added to a 2 mL tube with 500 mg glass beads and 0.2–0.5 g of sample. The mixture was vortexed for 1–5 min; then, 80 μL Buffer DS was added and mixed. Samples were incubated at 70 °C for 10 min with gentle inversion. After centrifugation at 13,000× *g* for 5 min, 600 μL of the supernatant was transferred to a new tube, mixed with 200 μL Buffer SP2 and 100 μL HTR reagent, followed by incubation on ice for 5 min and another centrifugation at 13,000× *g* for 5 min. Subsequently, 400 μL of the supernatant was mixed with 40 μL magnetic beads and 450 μL binding buffer, incubated for 2 min at room temperature, and then subjected to magnetic separation. Beads were washed once with 500 μL binding buffer and twice with 1000 μL Buffer PHB. After air drying, DNA was eluted with 50 μL elution buffer at 65 °C for 10–15 min.

The fungal ITS1 region was amplified using primers ITS1F (5′-CTTGGTCATTTAGAGGAAGTAA-3′) and ITS1R (5′-GCTGCGTTCTTCATCGATGC-3′), with a 7-base barcode for multiplexing. PCR reactions (25 μL) contained 14.75 μL sterile water, 5 μL 5× buffer (Roche), 2 μL of each dNTP (2.5 mM), 1 μL of each primer (10 μM), 1 μL template DNA, and 1 U of Q5 High-Fidelity DNA Polymerase (New England Biolabs, Ipswich, MA, USA). Thermocycling: 98 °C for 5 min; 30 cycles of 98 °C for 30 s, 55 °C for 30 s, 72 °C for 45 s; and a final extension at 72 °C for 5 min.

PCR products were purified with VAHTS™ DNA Clean Beads (Vazyme, Nanjing, China) and quantified using the Quant-iT PicoGreen dsDNA Assay Kit (Invitrogen, Carlsbad, CA, USA). Equimolar amplicons were pooled and sequenced on an Illumina NovaSeq 6000 platform (2 × 250 bp) at Personalbio (Shanghai, China).

### 2.3. Endophytic Fungal Diversity Analysis

Microbial community bioinformatics analyses were performed using QIIME2 version 2019.4 with minor modifications according to the official tutorial (https://docs.qiime2.org/2019.4/tutorials/, accessed on 21 September 2024) [16]. Raw sequence data were demultiplexed using the demux plugin, followed by primer trimming with the cutadapt plugin [17]. Quality filtering, denoising, merging of paired-end reads, and chimera removal were conducted using the DADA2 plugin [18]. Amplicon sequence variants (ASVs), excluding singletons, were aligned using MAFFT software (v7.520) [19]. Alpha diversity indices, including Chao1 richness estimator [20], observed species count, Shannon diversity index [21], and Simpson diversity index [22], were calculated using the diversity plugin. Beta diversity metrics [16] were also estimated. Taxonomic classification of ASVs was assigned using the feature-classifier plugin’s classify-sklearn naïve Bayes classifier against the UNITE database version 8.0 [23]. The sequence counts per sample after normalization are presented in the Supplementary Materials.

Sequence data analyses were primarily conducted using QIIME2 (v2019.4) and R (version 3.2.0). Within QIIME2, alpha diversity metrics at the ASV level (Chao1 richness, observed species, Shannon, and Simpson indices) were computed and visualized using boxplots. Rank abundance curves of ASVs were generated to compare richness and evenness among samples. Beta diversity analysis based on the Jaccard distance metric was performed to assess microbial community structural variation between samples. Visualization was conducted using principal coordinate analysis (PCoA), non-metric multidimensional scaling (NMDS), and unweighted pair group method with arithmetic mean (UPGMA) hierarchical clustering [24]. Additionally, principal component analysis (PCA) was performed based on genus-level compositional profiles [24].

Venn diagrams illustrating shared and unique ASVs among samples or groups, based on ASV presence/absence regardless of relative abundance, were generated using the R package (version 1.7.3) “VennDiagram” [25]. Linear discriminant analysis effect size (LEfSe) with default parameters was applied to identify taxa with significantly different abundances between groups [16]. Processing and analysis of endophytic fungal diversity data were conducted using the bioinformatics cloud platform provided by Shanghai Personalbio Technology Co., Ltd. (Shanghai, Chian, https://www.genescloud.cn, accessed on 21 March 2024).

All raw sequencing data have been deposited in the Sequence Read Archive (SRA) of the National Center for Biotechnology Information (NCBI) under accession number PRJNA1118471. Sampling site maps illustrating the collection locations of various alpine species were created using ArcGIS version 10.4 software [26].

Using ArcGIS 10.4 [26], a schematic diagram representing the collection points of various highland species was created. The relationship between the alpha diversity indices of different altitude groups and the average annual precipitation and temperature was plotted using OriginPro 2020b [27], with fitting curves illustrating these relationships. Autocorrelation analyses and principal component analysis (PC1) of the 19 climate factors were conducted using the R package “corrplot” [15]. Pearson correlation coefficients were calculated to exclude highly correlated climate variables, with the correlation threshold set at 0.8 [28]. Based on the calculation, four climate factors were selected for further fitting with the alpha diversity index: (i) temperature seasonality (bio4), (ii) mean temperature of coldest quarter (bio11), (iii) precipitation seasonality (bio15), and (iv) precipitation of the coldest quarter (bio19).

## 3. Results

### 3.1. Analysis of Endophytic Fungal Diversity

This study generated a total of 2,678,941 high-quality sequencing reads from the roots, stems, and leaves of four *C. lipskyi* populations across three regions, with the majority of reads ranging in length from 200–350 bp. The dilution curve, along with sequencing depth estimates, indicates that the sequencing depth is sufficient to capture the diversity of most fungi present in the samples. A total of 737 fungal ASVs were detected across all cloned libraries. The Venn diagrams illustrate the common and unique ASVs among the four samples, visualizing the ASV composition of each sample (Supplementary Figure S1). Four ASVs were shared among the endophytic fungal communities of the four *C. lipskyi* populations (T1, T2, T3, and T4) across the three regions, while 104, 125, 225, and 283 ASVs were unique to T1, T2, T3, and T4, respectively.

**Figure 2:**
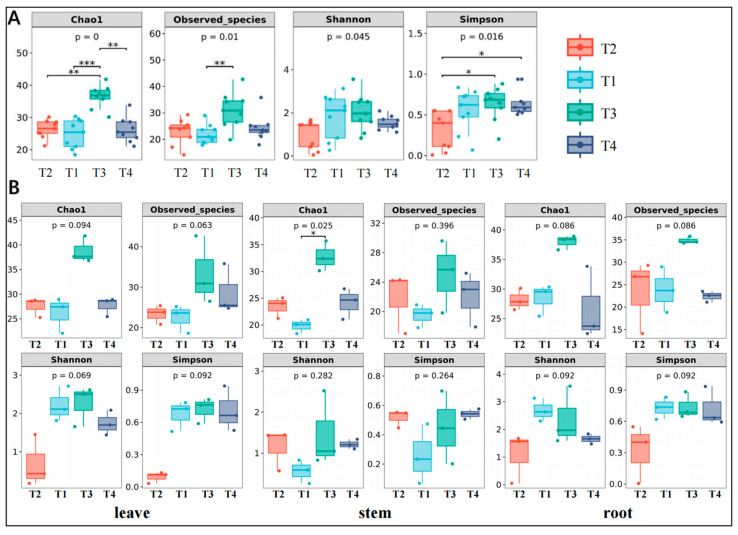
Alpha diversity index graph: (**A**) alpha diversity analysis of endophytic fungi in the whole plant; (**B**) analysis of alpha diversity of endophytic fungi in roots, stems, and leaves (* *p* < 0.05, ** *p* < 0.01, *** *p* < 0.001).

The analysis of alpha diversity using the Chao1 and observed species indices revealed significant variation in species richness across different altitudes, with the T3 group (2885.10 m) exhibiting higher species richness when compared to the other three altitude groups (T1: 2734.00 m, T2: 2687.00 m, T4: 3210.37 m). The lower-altitude group (T2) displayed lower Shannon and Simpson indices (Figure 2A). In contrast, higher altitudes (particularly the T3 group) exhibited significantly greater Shannon and Simpson indices.

The overall indices for the aboveground parts (stems and leaves, Figure 2B) were generally lower at the lowest and highest altitude groups but higher at intermediate altitudes, consistent with the overall alpha diversity trend in the plants. However, the alpha diversity indices for the underground parts (roots) did not show significant changes with increasing elevation. For the leaf tissues, the *p*-values for both the Chao1 and observed species indices were slightly elevated (0.094 and 0.063, respectively) for the T3 and T4 groups at higher elevations, but lower in the T2 group. For the Chao1 and observed species indices in the stem tissues, the T4 at higher elevation showed slightly greater species richness compared to other elevation groups, with a significant difference in the Chao1 index between the T3 and T4 (*p* = 0.025). The *p*-values for the Shannon and Simpson indices (0.282 and 0.264, respectively) were not significantly different across elevations. The Chao1 and observed species indices for the roots indicate that differences in species richness of endophytic fungi between elevations were not significant (*p* = 0.025 and *p* = 0.396). Nonetheless, the diversity indices for the higher-elevation groups (T3 and T4) were slightly higher than those of T2. The Shannon and Simpson indices also showed that changes in evenness and diversity of endophytic fungi in the roots across different elevations were not significant.

**Figure 3:**
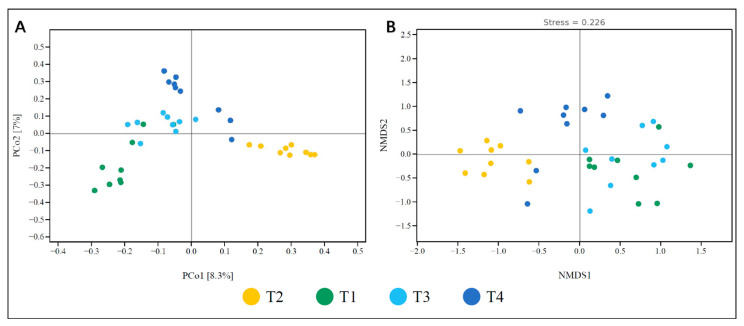
Beta diversity index graph: (**A**) distance matrix and PCoA analysis; (**B**) non-metric multidimensional scaling analysis.

The results of the principal coordinates analysis (PCoA) (Figure 3) indicated relatively small differences in endophytic fungal community composition among different *C. lipskyi* samples. The first two principal coordinates (PCoA1 and PCoA2) accounted for 8.3% and 7.0% of the total variance, respectively. Non-metric multidimensional scaling (NMDS) further revealed differences in fungal community diversity among samples, with the x- and y-axes representing NMDS1 and NMDS2, respectively (Figure 3). The reliability of the NMDS results was assessed using a stress value of 0.226, which indicates a moderate level of explanatory power and an acceptable goodness of fit. In the NMDS ordination space, samples from population T2 were closely clustered, as were samples from T1, suggesting that the endophytic fungal communities within each of these populations were relatively homogeneous. The environmentally conserved habitats of *C. lipskyi* likely explain the limited variance captured by the first two principal coordinates (PCoA1 and PCoA2). The endophytic fungal community in population T2 was markedly distinct from those in T1, T3, and T4, while T1 and T4 exhibited notably similar assemblages. This pattern underscores the considerable influence of altitudinal variation on fungal community structure and diversity.

### 3.2. Comparison of the Composition of Endophytic Fungal Communities

**Figure 4:**
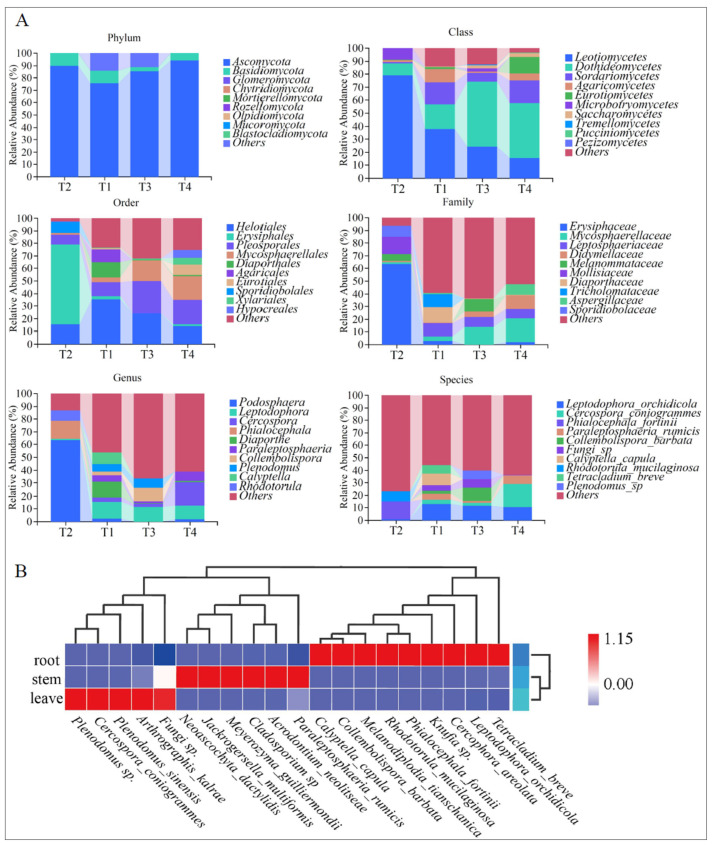
Taxonomic composition and species composition analysis: (**A**) analysis of endophytic fungal species in sampled plants; (**B**) heatmap of species composition of endophytic fungi in roots, stems, and leaves of sampled plants (The red to blue gradient in the figure represents high to low abundance).

All identified endophytic fungi belonged to three major phyla: Ascomycota (average relative abundance: 86.28%), Basidiomycota, and Glomeromycota (Figure 4A). At the class level, the top ten classes by relative abundance are Leotiomycetes, Dothideomycetes, Sordariomycetes, Agaricomycetes, Eurotiomycetes, Microbotryomycetes, Saccharomycetes, Tremellomycetes, Pucciniomycetes, and Pezizomycetes. At the order level, the ten most abundant orders are Helotiales, Erysiphales, Pleosporales, Mycosphaerellales, Diaporthales, Agaricales, Eurotiales, Sporidiobolales, Xylariales, and Hypocreales. At the family level, the ten most abundant families are Erysiphaceae, Mycosphaerellaceae, Leptosphaeriaceae, Didymellaceae, Melanommataceae, Mollisiaceae, Diaporthaceae, Tricholomataceae, Aspergillaceae, and Sporidiobolaceae. At the genus level, the ten most abundant genera were *Leptodontidium*, *Cercospora*, *Phialocephala*, *Diaporthe*, *Paraleptosphaeria*, *Collembolispora*, *Plenodomus*, *Calyptella*, and *Rhodotorula*. At the species level, the ten most abundant species are *Leptodophora orchidicola* (Sigler & Currah) Koukol & Maciá-Vicente, *Cercospora coniogrammes* Crous & R.G. Shivas, *Phialocephala fortinii* C.J.K. Wang & H.E. Wilcox, *Paraleptosphaeria rumicis* (Quaedvl., Verkley & Crous) Voglmayr, *Collembolispora barbata* Marvanová, Pascoal & Cássio, *Calyptella capula* (Holmsk.) Quél., *Rhodotorula mucilaginosa* (A. Jörg.) F.C. Harrison, and *Tetracladium breve* A. Roldán.

### 3.3. Comparison of Species Composition Variability

The composition of endophytic fungal species in different organs of *C. lipskyi*. exhibited significant variation. In the roots, *Tetracladium breve*, *Leptodophora orchidicola*, and *Cercophora areolata* were found at higher abundance but showed lower levels in stems and leaves. This suggests that root-associated fungal communities are strongly adapted to the soil environment, likely due to their close association with root physiological functions such as nutrient and water uptake, as well as interactions with rhizosphere microorganisms.

In contrast, fungal communities in the stems and leaves were more similar to each other but still displayed distinct organ-specific characteristics. For instance, *Phialocephala fortinii* and *Cercospora coniogrammes* were more abundant in stems, but less so in the roots and leaves. Species such as *Phialocephala fortinii* and *Rhodotorula mucilaginosa* showed significantly higher abundance in stems, possibly reflecting adaptations to stem-specific structural or physiological conditions, indicating that the microenvironments of different plant organs selectively influence fungal colonization.

Previous studies have also shown that species such as *Calyptella capula* and *Cladosporium* sp. are more abundant in leaves, which is likely related to the unique exposure of leaf tissues to environmental factors. Leaves are typically subject to UV radiation, desiccation, and pathogen attack. As a result, the leaf-associated fungal community tends to exhibit greater environmental adaptability. These fungi may assist the host plant by regulating photosynthesis, reducing water loss, and enhancing resistance to pathogens [29].

### 3.4. The Correlation Between the Diversity of Endophytic Fungi in Plants and Elevation

**Figure 5:**
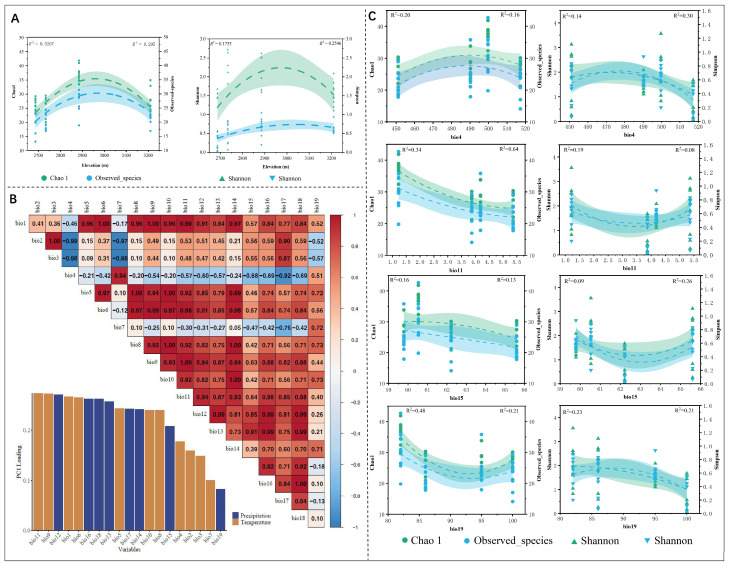
Diversity index fitting and autocorrelation analysis: (**A**) diversity index fitting diagram with altitude; (**B**) analysis of the autocorrelation of 19 biological data points and the PC1 analysis graph; (**C**) fitting curves of temperature seasonality (bio4), mean temperature of coldest quarter (bio11), precipitation seasonality (bio15), precipitation of the coldest quarter (bio19) with respect to the alpha diversity index. The curve fitting was performed using a binomial equation, with the confidence interval set at 90%.

In the altitude range of 2600–2900 m, the diversity indices (Chao1, observed species, Simpson, and Shannon indices, Figure 5A) of endophytic fungi in *C. lipskyi* showed a positive correlation with average annual precipitation and a negative correlation with average annual temperature. However, above 3000 m, the diversity indices of endophytic fungi associated with the alpine medicinal plant displayed a negative correlation with average annual precipitation and a positive correlation with average annual temperature. The analysis of alpha diversity, particularly through the Chao1 and observed species indices, revealed significant differences in species richness across various altitudes. Notably, the T2 group (2687 m) exhibited a lower species richness when compared to that of other altitude groups (T1: 2734 m, T3: 2885.10 m, and T4: 3210.37 m).

PC1 was used to select the climate factors bio4, bio11, bio15, and bio19 for fitting with the alpha diversity indices (including Chao1, observed species, Shannon, and Simpson indices) (Figure 5B). The results showed that as the value of temperature seasonality (bio4) increased, the diversity index initially increased and then decreased (Figure 5C). The highest diversity index was observed when the bio4 value ranged from 490 to 500, corresponding to an altitude range of 2885–3210 m (Table 1). In contrast, the average temperature of the coldest quarter (bio11) was negatively correlated with Chao1 and observed species indices, with the highest diversity index observed at a bio11 value of 1.22 °C (altitude 2885.1 m). However, for the Shannon and Simpson indices, a trend of initial decline followed by an increase was observed, with the highest diversity index occurring at a bio11 value of 1.22 °C (altitude 2885.1 m). Further analysis revealed that precipitation during the warmest quarter (bio15) was negatively correlated with Chao1 and observed species indices, with the highest diversity index observed at a bio15 value of 60.53 mm (altitude 2885.1 m). Shannon and Simpson indices exhibited a trend of initial decline followed by an increase, with the highest diversity index observed at a bio15 value of 59.78 mm (altitude 3210 m). Precipitation during the coldest quarter (bio19) showed an initial decline followed by an increase in Chao1 and observed species indices, with the highest value observed at a bio19 value of 86.00 mm (altitude 2687 m). In contrast, Shannon and Simpson indices exhibited an initial increase followed by a decrease, with the highest diversity index observed at a bio19 value of 86.00 mm (altitude 2687 m). In the fitting results of each group, bio11 showed the highest R^2^ values with Chao1 and observed species indices, which were 0.34 and 0.64, respectively.

## 4. Discussion

### 4.1. Analysis of Species Composition in Taxonomy

The analysis of the taxonomic composition revealed that the endophytic fungi in the Gaoligong Mountains *C. lipskyi* predominantly belong to the phylum Ascomycota, which is consistent with previous research findings [30]. Among these groups, the Phylum Ascomycota exhibited the highest abundance, establishing its status as the unequivocal dominant community. Ascomycota was also reported as the predominant endophytic phylum associated with medicinal plants in India [31]. Additionally, Ascomycota has been identified as the predominant endophytic phylum in the medicinal plant *Ocimum sanctum* [32]. At the class level, there was a significant increase in Dothideomycetes with rising altitude, while the abundance of Leotiomycetes significantly decreased [14,33]. Other classes, such as Agaricomycetes, Eurotiomycetes, and Sordariomycetes, showed distinct distribution patterns across different populations. Global diversity studies on plant and soil fungi have revealed that Sordariomycetes and Agaricomycetes are more abundant in soil and root environments [34]. In contrast, Eurotiomycetes are predominantly found as parasites on plant organs, such as leaves [35]. The distribution of these groups is influenced by factors such as climate, soil type, and vegetation characteristics [36,37]. At the order level, Pleosporales and Mycosphaerellales showed a significant increase with altitude, whereas Helotiales displayed a notable trend of initially increasing and then decreasing with elevation. This pattern is consistent with previous research findings [38]. At the family level, Mycosphaerellaceae significantly increased with altitude, while Leptosphaeriaceae was not found in populations below 2700 m. At the genus level, populations above 2700 m exhibited significantly higher levels of *Leptodophora* compared to T2, with *Cercospora* reaching its highest abundance in populations above 3200 m, and this genus was not detected in the populations below 2700 m (T2). *Phialocephala* was only found in the population below 2700 m (T2). Among them, the genus *Cercospora*, belonging to the phylum Ascomycota, is typically regarded as a group of plant pathogens capable of causing various diseases such as leaf spot in crops. However, recent studies indicate that certain strains of *Cercospora* can produce secondary metabolites, including polysaccharides, flavonoids, total triterpenes, and total phenols, which hold potential application value in the production of bioactive medicinal compounds [38]. The new compound phialoyxinone B, extracted from *Phialocephala* sp. YUD18001, isolated from the rhizosphere soil of *Gastrodia elata*, exhibits in vitro cytotoxic activity against human cancer cells [39].

### 4.2. Analysis of Variability in Species Composition and Its Potential Medicinal Value

The endophytic fungal community composition varied significantly among different organs of *C. lipskyi*. In the heatmap visualization (Figure 4B), red indicates areas of high relative abundance, whereas blue indicates lower abundance. In the roots, *Tetracladium breve*, *Leptodophora orchidicola*, and *Cercophora areolata* were present at relatively high abundances, while their representation was much lower in the stems and leaves. This suggests that root-associated fungal communities are strongly adapted to the soil microenvironment, likely due to the physiological roles of roots in nutrient and water uptake and their close interactions with rhizosphere microorganisms. By contrast, fungal communities in stems and leaves appeared more similar to each other, yet still exhibited organ-specific differences. For example, *Phialocephala fortinii* and *Cercospora coniogrammes* were more abundant in stems but had relatively low abundance in roots and leaves. Certain stem-associated taxa, such as *Phialocephala fortinii* and *Rhodotorula mucilaginosa*, were significantly enriched, potentially reflecting structural and physiological characteristics of the stem. These findings suggest that the microenvironments of different plant organs are selective filters for the colonization of specific fungal taxa.

Studies on endophytic fungi and their secondary metabolites have shown that some dominant fungal taxa in the roots of *C. lipskyi* exhibit considerable medicinal properties and potential. Among them, *Phialocephala fortinii* and *Rhodotorula mucilaginosa*, which exhibited high relative abundance in the roots, have been recognized for their bioactive properties. *Phialocephala fortinii* is known to interact with host plants and influence their metabolic pathways. Research has shown that *P. fortinii* can modulate gene expression and metabolic processes in the host plant [40], and it is capable of producing various enzymes that benefit the host when colonizing root tissues. Likewise, *Rhodotorula mucilaginosa*, a red yeast widely distributed in natural environments, has attracted attention for its ability to produce functional bioproducts such as natural carotenoids, lipids, and enzymes [41]. These compounds are of considerable interest for applications in the fields of food, medicine, cosmetics, and bioenergy [42]. In the stems of *Saussurea medusa Maxim*, *Cladosporium* sp. and *Meyerozyma guilliermondii* were found in relatively high abundance. Fungi belonging to the genus *Cladosporium* have been recognized as valuable microbial resources with medicinal potential. They are known to produce a variety of biologically active secondary metabolites with antitumor, antimicrobial, and antioxidant properties. For instance, two naphthoquinone compounds—anhydrofusarubin and methyl ether of fusarubin—isolated from a *Cladosporium* species have demonstrated cytotoxic activity against human leukemia cells (K-562) as well as significant antibacterial effects against certain bacterial strains [43]. In addition, *Cladosporium* species. has been found to produce other bioactive compounds with antifungal, antiviral, and immunomodulatory activities [44,45]. The presence of these functionally specialized endophytic fungi likely influences the metabolic pathways of the endophytic community within *S. medusa*, contributing to the enrichment of biosynthetic routes associated with pharmacologically active compounds.

### 4.3. Mechanisms of Environmental Factors Influencing the Diversity of Plant Endophytic Fungi

The diversity of endophytic fungi is always significantly influenced by environmental factors, with altitude being a crucial driving force [46]. Existing research indicates that as altitude increases, variations in environmental factors such as temperature, humidity, soil characteristics, and plant community composition can significantly affect the diversity, abundance, and distribution patterns of endophytic fungi [47].

Scatter plots and linear fitting analyses revealed that, within the elevation range of 2600–2900 m, the diversity indices of endophytic fungi in the roots, stems, and leaves of *C. lipskyi* (Chao1, observed species, Simpson, and Shannon indices) were positively correlated with elevation and annual precipitation, but negatively correlated with annual temperature [48]. When combined with bioclimatic variables (bio4, bio11, bio15, and bio19), it was found that smaller seasonal temperature variation and seasonal precipitation variation occurred in the *C. lipskyi* habitat. From our observation, the more stable the climatic factors of the habitat, the more favorable for the reproduction and survival of endophytic fungi. Certain cold-tolerant fungal species can adapt to high-altitude environments and may even achieve higher reproductive success at these elevations. Lower temperatures and precipitation in the coldest quarter also provide a favorable environment for the survival of some endophytic fungi, which helps explain the increased species richness and community diversity observed at higher elevations. These findings align with existing research [49,50]. However, at elevations exceeding 3000 m, the diversity indices of endophytic fungi in *C. lipskyi* showed a negative correlation with elevation and annual precipitation, but a positive correlation with annual temperature. As elevation increases, lower temperatures, larger diurnal temperature fluctuations, and harsher environmental conditions limit the growth of certain fungi, ultimately restricting fungal abundance and species richness. This result is consistent with previous studies [51]. Interestingly, the Shannon and Simpson indices at sampling sites above 3000 m were higher than those at the lowest elevation sites, indicating that with increased elevation, as well as higher average temperatures and precipitation in the coldest quarter, high-altitude regions exhibit more complex community structures and more even species distributions. This finding further supports the environmental filtering hypothesis, which suggests that under harsher environmental conditions, such as those at high altitudes, ecosystems tend to select species with stronger adaptability, resulting in more stable communities [52]. The more complex community structures in high-altitude regions may reflect the co-evolutionary process between fungi and plants, as well as their adaptive mechanisms in response to altitude changes.

Alpha diversity analysis revealed significant differences in the fungal endophytic community among leaves, stems, and roots across different populations (Figure 2B). This discrepancy may be attributed to the physiological functions of different plant organs and the specific microenvironmental conditions they encounter. Based on our observation, various plant organs may harbor different microbial community compositions due to their varying exposure durations and intensities to external environmental factors. Leaves are typically more directly influenced by external conditions such as humidity, light, and temperature [53], while the fungal communities associated with stems and roots are more significantly regulated by the internal conditions of the host plant [54]. The aboveground parts (stems and leaves) exhibited a trend where the index initially increased with the elevation of the sampling points, followed by a subsequent decline. This pattern is similar to the overall alpha diversity analysis results of the plant species. In the leaf region, elevation may have a certain impact on species richness, as higher altitudes provide more habitat niches and favorable ecological environments for endophytic fungi. The diversity of endophytic fungi within leaf tissue may be influenced by factors such as microclimatic conditions, which fluctuate with changes in elevation [55]. At the stem base, the diversity of endophytic fungal species increases with rising altitude; however, the species richness index of endophytic fungi at the root level does not show significant variation. This may be attributed to the fact that root-associated fungi primarily depend on the soil and the rhizosphere microenvironment of the host plants, resulting in a relatively stable composition of endophytic fungal species within the root tissues [56].

The results from the PCoA and NMDS analysis of beta diversity (Figure 3) also confirmed the differences in endophytic fungal communities between low and high-altitude regions. When combined with alpha diversity, our findings suggest that endophytic fungal community diversity increases with altitude, whereas lower-altitude regions exhibit relatively uniform communities, indicating that the diversity of endophytic fungal communities is strongly influenced by environmental gradients [57]. Furthermore, plant communities in the same region may share similar fungal communities, but local environmental differences can still lead to some degree of diversity variation [58].

## 5. Conclusions

This study represents the first report on the composition and diversity of endophytic fungal communities in the alpine plant *C. lipskyi* and the potential changes in fungal composition and diversity under the stress of high-altitude cold environments. Furthermore, it also highlights the medicinal potential of endophytic fungi in various plant organs, providing a theoretical basis for future research on the diversity and potential applications of endophytic fungi in *C. lipskyi* from the Gaoligong Mountains region. However, due to the lack of mature transplantation cases for *C. lipskyi* and the random nature of field sampling, the current results may not fully represent the endophytic fungal diversity in the region. Future studies will continue to explore the diversity and potential applications of endophytic fungi in *C. lipskyi* from Gaoligong Mountains and its surrounding areas.

## Figures and Tables

**Table 1:** Details of sampling of *Carpesium lipskyi* in the Gaoligong Mountains in Yunnan, China. bio4: temperature seasonality, bio11: mean temperature of coldest quarter, bio15: precipitation seasonality, bio19: precipitation of the coldest quarter.

ID	Collection Site	Longitude	Latitude	Altitude (m)	Growth Stage	Plant Height (cm)	Bio4 (standard Deviation × 100)	Bio11 (°C)	Bio15 (mm)	Bio19 (mm)
T2	Shiyueliang Township, Fugong, Nujiang Prefecture, Yunnan Province	98°46′49.82”	27°9′55.02”	2687.00	florescence	57	451.05	3.87	62.22	100.00
T1	Panma Town, Lushui, Nujiang Prefecture, Yunnan Province	98°40′17.11”	25°59′0.78”	2734.00	florescence	54	517.21	5.37	65.60	86.00
T3	PiheNuZu Township, Fugong, Nujiang Prefecture, Yunnan Province	98°59′27.06”	26°33′48.34”	2885.10	florescence	61	499.68	1.22	60.53	82.00
T4	PiheNuZu Township, Fugong, Nujiang Prefecture, Yunnan Province	98°58′37.73”	26°32′52.17”	3210.37	florescence	57	490.17	4.18	59.78	95.00

## Data Availability

The data presented in this study are openly available in the Sequence Read Archive (SRA) of the National Center for Biotechnology Information (NCBI) at https://www.ncbi.nlm.nih.gov/bioproject/?term=%20PRJNA1118471 accessed on 24 July 2025, reference number PRJNA1118471.

## Supplementary Materials

The following supporting information can be downloaded at: https://www.mdpi.com/article/10.3390/jof11100704/s1, Figure S1: Dilution curves of the ITS1 region sequences of the endophytic fungi of the sample plants and the Venn diagram of the ASV of the endophytic fungi of the sample plants; Table S1: Statistics of sequences from all *C. lipskyi* samples after filtering out low-quality and chimeric reads based on DADA2; Table S2: Codes of bioclimatic variables and their corresponding names (Codes of bioclimatic variables according to WorldClim database (version 2.0, http://worldclim.org/version2, accessed 10 November 2021).
