# Supplementary material for: Endophytic Fungal Diversity in Carpesium lipskyi from the Gaoligong Mountains, Yunnan, China
Source: J Fungi (Basel). 2025 Sep 28;11(10):704. doi: 10.3390/jof11100704 (PMC12565673; doi:10.3390/jof11100704)
Supplement: Supplementary file 1 [file jof-11-00704-s001.zip › jof-3809903-supplementary.pdf]

Supplementary Materials

1. Supplementary Figures and Tables

1.1 .Supplementary Figure

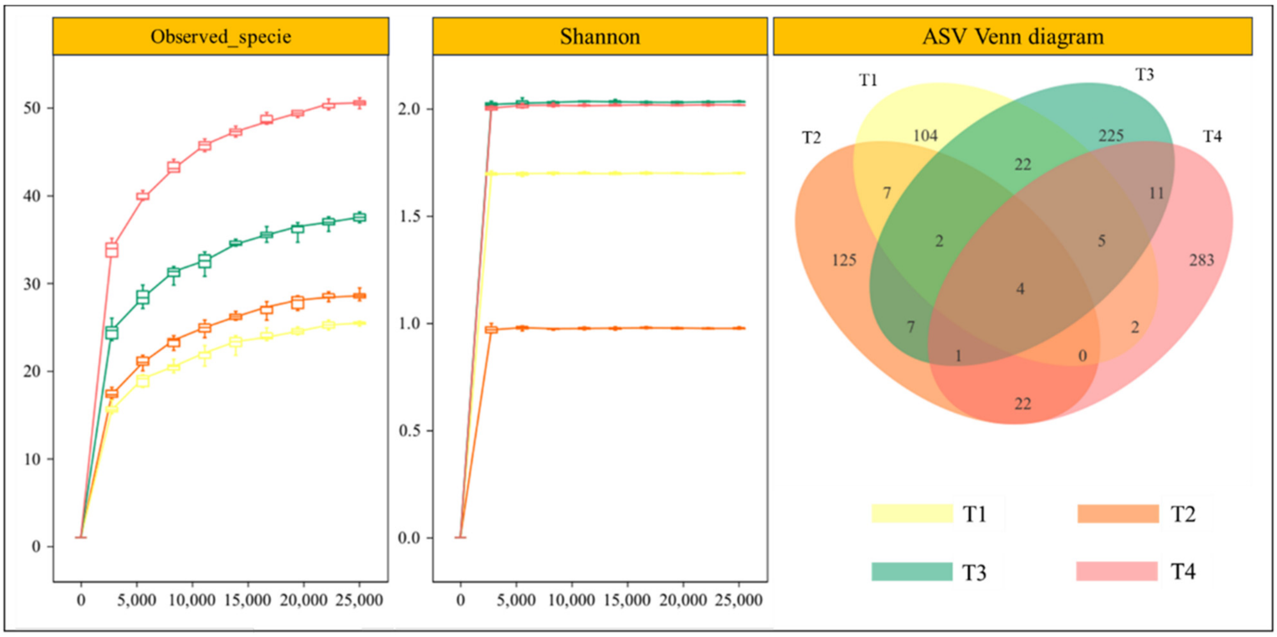

Supplementary Figure S1. Dilution curves of the ITS1 region sequences of the endophytic fungi of the sample plants and the Venn diagram of the ASV of the endophytic fungi of the sample plants.

1.2. Supplementary Tables

Supplementary Table S1. Statistics of sequences from all *C. lipskyi* samples after filtering out low-quality and chimeric reads based on DADA2.

SampleID	Input	Filtered	Denoised	Merged	Non-chimeric	Non-singleton
T1_1_g	78178	66670	66613	65660	62013	62013
T1_1_j	64898	61536	61510	61168	61045	61045
T1_1_y	97423	90629	90504	89236	76743	76742
T1_2_g	75532	63640	63613	62337	59643	59643
T1_2_j	82094	77019	76996	76489	75982	75982
T1_2_y	80769	74821	74785	64830	63185	63185
T1_3_g	70512	65036	65017	64833	63898	63898
T1_3_j	69420	65271	65194	64747	60997	60997
T1_3_y	83277	77777	77753	77654	73297	73297
T2_1_g	120055	101982	101963	101399	96718	96718
T2_1_j	90594	72640	72471	71702	65531	65531
T2_1_y	108125	81418	81387	80747	79231	79231
T2_2_g	209375	109773	109689	109192	107737	107737
T2_2_j	93052	69635	69584	69445	63396	63395
T2_2_y	94578	60311	60255	59873	58742	58742
T2_3_g	94474	87547	87471	87104	84726	84725
T2_3_j	91748	72336	72150	71705	71705	71705
T2_3_y	93594	50994	50960	50581	50575	50575
T3_1_g	359573	158343	158084	64381	63725	63721
T3_1_j	101098	91951	91771	90098	88566	88566
T3_1_y	114101	107583	107492	106430	93804	93803
T3_2_g	86937	71022	70991	70591	66128	66128
T3_2_j	99360	94288	94111	92869	81426	81426
T3_2_y	92271	85106	85053	84782	80408	80408
T3_3_g	83144	71984	71970	71551	67590	67590
T3_3_j	90844	86322	86068	85520	79161	79161
T3_3_y	102285	95058	95026	94355	91621	91621
T4_1_g	89250	83305	83260	83067	82183	82183
T4_1_j	324673	32766	32316	29966	26382	26382
T4_1_y	335860	75085	74422	70964	61511	61511
T4_2_g	95620	86891	86746	86673	86266	86266
T4_2_j	84525	77880	77829	77689	76667	76667
T4_2_y	101972	94873	94821	94340	94160	94160
T4_4_g	90403	81353	81216	80857	80339	80339
T4_4_j	92250	86541	86415	85929	83832	83832
T4_4_y	111172	103528	103458	103312	100008	100008

Supplementary Table S2. Codes of bioclimatic variables and their corresponding names (Codes of bioclimatic variables according to WorldClim database (version 2.0, http://worldclim.org/version2, accessed 10 November 2021).

Code	Bioclimatic Variable
**BIO1**	Annual mean temperature (°C)
**BIO2**	Mean diurnal range (mean of monthly, maximum temperature—minimum temperature) (°C)
**BIO3**	BIO3 Isothermality (BIO2/NIO7) (×100)
**BIO4**	Temperature seasonality (standard deviation _100)
**BIO5**	Maximum temperature of warmest month (°C)
**BIO6**	Minimum temperature of coldest month (°C)
**BIO7**	Temperature annual range (BIO5-BIO6) (°C)
**BIO8**	Mean temperature of wettest quarter (°C)
**BIO9**	Mean temperature of driest quarter (°C)
**BIO10**	Mean temperature of warmest quarter (°C)
**BIO11**	Mean temperature of coldest quarter (°C)
**BIO12**	Annual precipitation (mm)
**BIO13**	Precipitation of wettest month (mm)
**BIO14**	Precipitation of driest month(mm)
**BIO15**	Precipitation seasonality (coefficient of variation) (mm)
**BIO16**	Precipitation of wettest quarter (mm)
**BIO17**	Precipitation of driest quarter (mm)
**BIO18**	Precipitation of warmest quarter (mm)
**BIO19**	Precipitation of coldest quarter (mm)
